# Perioperative acute myocardial infarction in patients after non-cardiac surgery in China: Characteristics and risk factors

**DOI:** 10.1097/MD.0000000000016929

**Published:** 2019-08-23

**Authors:** Xiaoxin Zhou, Lingke Chen, Zhongxue Su, Yue Li, Mengyun Tu, Jie Xiao, Zhiying Pan, Diansan Su

**Affiliations:** a Department of Anesthesiology, Renji Hospital, School of Medicine, Shanghai Jiaotong University, Shanghai; b Department of Anesthesiology, The First Affiliated Hospital of Soochow University, Suzhou, Jiangsu, China.

**Keywords:** clinical characteristic, mortality, non-cardiac surgery, perioperative acute myocardial infarction

## Abstract

To examine the characteristics and short-term outcome of perioperative myocardial infarction (PMI), a single-center retrospective study was carried out. The electronic medical records of 278,939 patients aged 45 years or older who underwent non-cardiac surgery at Renji Hospital from January 2003 to December 2015 were screened based on diagnostic codes (ICD121, ICD121.0, ICD121.1, ICD121.2, ICD121.3, ICD121.4, or ICD121.9). The incidence and characteristics of PMI and mortality risk factors were analyzed after non-cardiac surgery. PMI was reported in 45 patients, with an incidence rate of 1.61 per 10,000 and a mortality rate of 75.6% (34/45). The PMI incidence rate increased significantly with age. The PMI incidence rate was the highest for vascular surgery (5.82 per 10,000 cases). PMI occurs mainly within 48 h of surgery, with most cases showing an onset in the general wards. Logistic analysis showed that the use of nitrates is the independent protective factor for the outcomes of patients with PMI. The incidence of PMI in non-cardiac surgery is approximately 2 of 10,000 in patients aged 45 years or older, and increased significantly with age. The use of nitrates might be helpful for their survival.

## Abstract 2

Supplemental Digital Content is available in the text

## 1 Introduction

Cardiovascular complications are the major contributors to morbidity and mortality following non-cardiac surgeries. Perioperative myocardial infarction (PMI) is one of the most severe complications and causes high mortality and prolonged hospital stays. In patients with PMI, the odds ratio (OR) was 10 (95% confidence interval [CI]: 7.8–12.9) for death, and a composite of cardiovascular complications has been found at 30 days.^[1]^

Millions of major non-cardiac procedures are performed in China annually. With the rapid development of medical care and technology, a booming number of elderly patients with multiple comorbidities places demands on surgery, which increases the risk for adverse cardiac events. Meanwhile, the amount of PMI data from China is limited. Moreover, due to its silent presentation, clinicians and the public remain unaware of the severity of PMI in China. The incidence and characteristics of PMI in patients undergoing non-cardiac surgery were investigated to alert clinical physicians about this silent killer and to provide insights into its prevention, diagnosis, and treatment.

## 2 Methods

This study was approved by the ethics committee of Renji Hospital, School of Medicine, Shanghai Jiaotong University (approval number: Renji[2018]200). This single-center study retrospectively screened the electronic and paper medical records of patients aged 45 years or older at Renji Hospital Affiliated to the Shanghai Jiaotong University School of Medicine. Following screening by diagnostic codes, PMI was reported in 45 patients. These patients were then divided into survival and mortality groups based on their outcomes within 30 days after the onset of PMI. The observation period lasted from admission to discharge.

### 2.1 Screening process

In total, 278,939 patients aged ≥45years undergoing non-cardiac surgery at our hospital from January 2003 to December 2015 were selected. The electronic medical records were available in the inquiry system set up in 2013. The medical records were reviewed independently by two clinicians, and if there were different opinions about the diagnoses, the final decision was made by a senior doctor. The records were screened by diagnostic codes (ICD121, ICD121.0, ICD121.2, ICD121.3, ICD121.4, or ICD121.9). The detailed screening process is described in supplementary Fig. 1.

### 2.2 Diagnostic criteria

The following conditions meet the diagnosis of PMI: the detection of an increase and/or a decline in the levels of a cardiac biomarker with at least one value above the 99th percentile upper reference limit and with at least one of the following:

1. symptoms of ischemia;

2. new or presumed new significant ST-segment and T wave changes or new left bundle branch block;

3. the development of pathological Q waves in the electrocardiogram (ECG);

4. imaging evidence of new loss of viable myocardium or new regional wall motion abnormality; and

5. the identification of an intracoronary thrombus by angiography or autopsy.^[2]^

The definition of intraoperative hypotension is mean arterial pressure < 60 mm Hg for ≥1 h or patients requiring the administration of vasoactive drugs to maintain a stable blood pressure. Intraoperative tachycardia is defined as a heart rate > 100 bpm that is sustained for ≥1 h.

Diagnostic criteria of chest pain were as follows:

1. sudden chest pain lasting for ≥30 min;

2. location of chest pain (mainly in the retrosternal area or that may radiate to the jaw, neck, arms, back, and epigastria);

3. quality of chest pain (described as tightness, pressure, or squeezing);

4. accompanied by ECG changes;

5. excluded surgery-related chest pain; and

6. chest pain with a coronary angiogram (if any).

### 2.3 Clinical data

Detailed clinical data, including age; sex; the presence of diabetes or hypertension; smoking status; a history of myocardial infarction (MI), stable angina, or heart failure; prior myocardial revascularization procedures; the use of cardiovascular medication; and the type of operation and anesthesia, were collected. The patients were followed up until hospital discharge, and information on recurrent unstable angina, MI, and death was obtained. The time and location during the onset of PMI, type of PMI, and complications after PMI were also recorded. In addition, the preoperative treatment, PMI treatment, and duration of intensive care unit (ICU) stay were recorded.

### 2.4 Statistical analysis

Statistical analyses were performed using the Statistical Package for the Social Sciences for Windows (version 22.0; SPSS Inc.; Chicago, IL, USA). The constituent ratios of many factors (e.g., age and sex) were analyzed in each group, and the PMI incidence rate was adjusted for the unbalanced factor. The groups formed based on sex were adjusted for age, and those formed based on age and surgery were adjusted for sex. The chi-square and Fisher's exact tests were performed to identify the difference between proportions. Student's *t* test was used to analyze continuous data, and the results are reported as mean ± standard deviation values. Rank data were compared using the Mann–Whitney *U* test and are reported as median (minimum, maximum) values. After performing univariate analysis, binary logistic regression analysis was performed to estimate the ORs of the independent risk factors. A *P*-value < .05 (two-sided) was considered statistically significant. Graphs were constructed on GraphPad Prism 5.0 (GraphPad Software Inc., La Jolla, CA, USA).

## 3 Results

### 3.1 PMI incidence increased with age and varied with the type of surgery

In total, 278,939 patients aged ≥ 45 years undergoing non-cardiac surgeries at our hospital from January 2003 to December 2015 were enrolled. Of the 278,939 patients, 7763 (27.83%%o) patients had complications of coronary heart disease and 45 had PMI (1.61 per 10,000). Of the 45 patients, only 11 patients survived (mortality rate of 75.6%).

**Figure 1:**
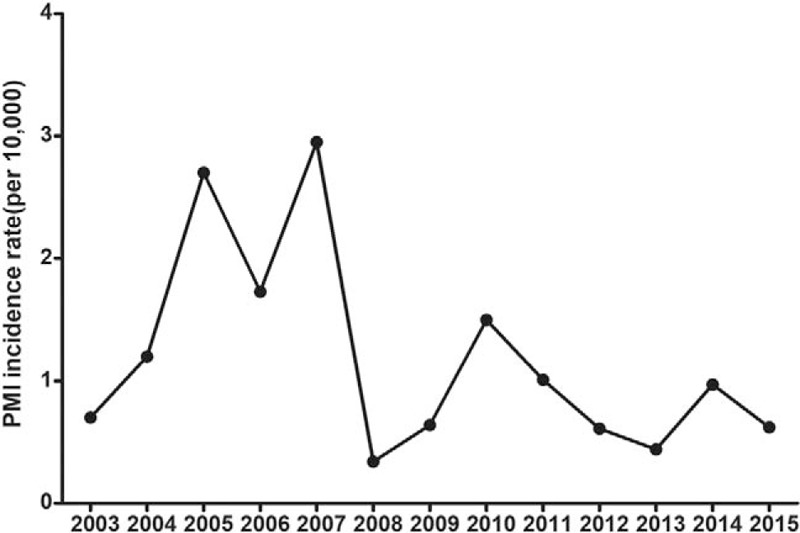
The PMI incidence in patients who underwent non-cardiac surgeries at Shanghai Renji Hospital from 2003 to 2015. PMI = perioperative myocardial infarction.

**Figure 2:**
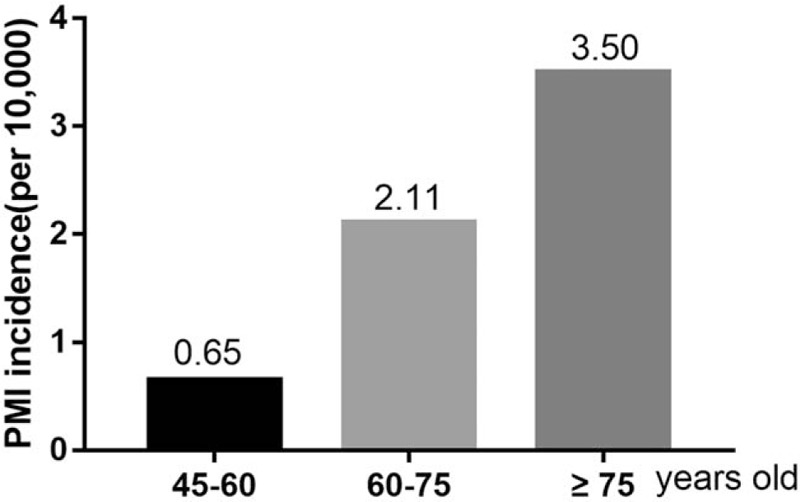
PMI incidence rate of patients based on age group. PMI = perioperative myocardial infarction.

The mean age of all 45 patients with PMI was 71.4 years (range: 45–90 years). Generally, the annual incidence of PMI showed a gradual decline (Fig. 1). The PMI incidence rate increased significantly with age (adjusted for sex): patients aged between 45 and 60 years, 0.65 per 10,000; patients aged between 60 and 75 years, 2.11 per 10,000; and patients older than 75 years, 3.50 per 10,000 (*P* < .001; Fig. 2 and Table 1).

**Table 1:**
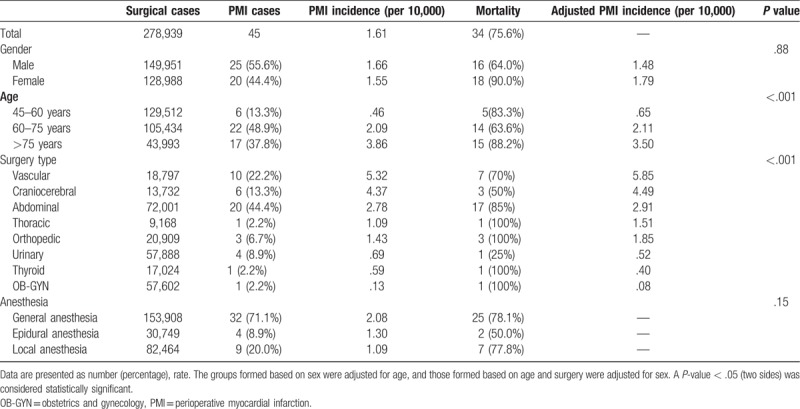
Perioperative myocardial infarction incidences.

The PMI incidence rate was verified with the type of surgery. Of the 45 PMI cases, 20 (44.4%) were of abdominal surgeries, 10 (22.2%) of vascular surgeries, 6 (13.3%) of craniocerebral surgeries, 4 (8.9%) of urological surgeries, 3 (6.7%) of orthopedic surgeries, and 1 case (2.2%) each of thoracic, thyroid, and obstetrics and gynecology surgeries. The PMI incidence rate was the highest for vascular surgery (5.85 per 10,000 cases, adjusted for gender), followed by craniocerebral surgery (4.49 per 10,000 cases, adjusted for gender) (Table 1).

Different anesthesia methods had no effects on the incidence of PMI. A total of 153,908 patients were administered general anesthesia, and PMI was reported in 32 patients, with an incidence of 2.08 per 10,000. Four cases of PMI were reported among 30,749 patients who were administered epidural anesthesia, resulting in an incidence of 1.30 per 10,000. Nine cases of PMI were confirmed in 82,464 patients who underwent local anesthesia, resulting in an incidence of 1.09 per 10,000 (*P* = .15, Table 1).

No sex-related difference was observed in terms of PMI incidence. The PMI incidence rate for males was 1.48 per 10,000 (25/149,951, adjusted for age) and that for females was 1.79 per 10,000 (20/128,988, adjusted for age) (Table 1).

### 3.2 PMI occurs mainly within 48 h of surgery and most patients had an onset in the general wards

PMI occurred in only one patient during the induction of anesthesia. PMI occurred in 27 (60%) cases within 48 h of surgery, while it occurred in 18 (40%) cases after 48 h. Moreover, the onset of PMI was detected later in the mortality group than in the survival group (*P* = .001; Table 2).

**Table 2:**
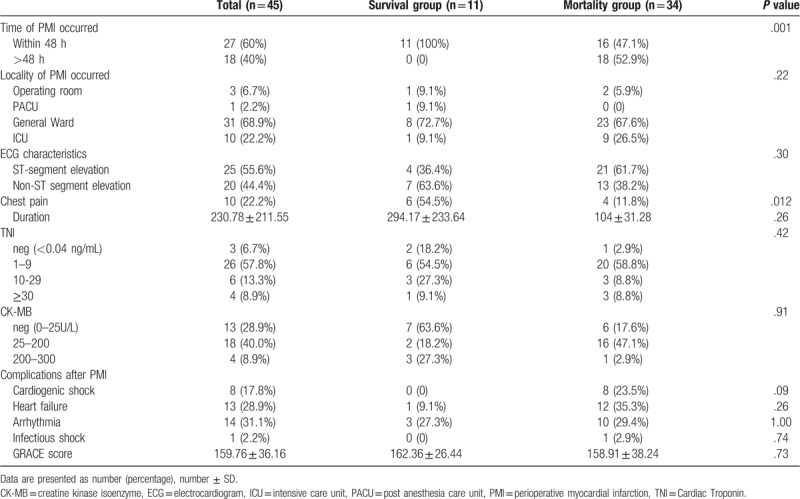
PMI characteristics.

Most PMI cases had an onset in the general wards; 3 (6.7%) PMI cases occurred in the operating rooms, 1 (2.2%) in the post-anesthesia care unit, 31 (68.9%) in the general wards, and 10 (22.2%) in the ICU. The location where PMI occurred had no difference between the patients in the survival and mortality groups (*P* = .22, Table 2).

### 3.3 Patients with PMI lack chief complaints

Chest pain is the major complaint of MI, accompanied by tightness in the chest, shortness of breath, and palpitations. However, in this study, only 10 patients had complaints of chest pain. The mean duration of chest pain was 230.78 min (range: 30–540 min) (Table 2).

The ECG features, myocardial enzyme profiles, and complications of PMI showed no differences in the two groups. Of all the 45 patients with PMI in this study, 20 (44.4%) had non-ST-segment elevation myocardial infarction (NSTEMI; type 2) and 25 (55.6%) had ST-segment elevation myocardial infarction (STEMI; type 1). The two PMI types were almost evenly distributed (Table 2).

### 3.4 Nitrates may have cardioprotective effects

The patients were divided into two groups according to their outcomes within 30 days following surgery. After performing univariate analysis, the following 6 factors (*P* < .05) were included in the binary logistic regression analysis: the time at which PMI occurred; whether the patients complained of chest pain; the application of statins, nitrates, and anticoagulants; and procedures of percutaneous coronary intervention (PCI) and coronary artery bypass grafting (CABG). The results showed that the use of nitrates may protect patients with PMI (OR: 0.04; 95% CI: 0.00–0.60) from death (*P* = .02). Nitrates can improve cardiac perfusion and decrease cardiac preload, so typically, nitrates are not used in patients with severe hypotension and cardiogenic shock. Considering the possible selection bias, we compared the severity of both groups using the Global Registry of Acute Coronary Events score (GRACE),^[3]^ there was no significant difference between the two groups, *P* = .73 (see Table 2).

### 3.5 Revascularization may still be the most effective way to treat PMI

All three patients undergoing revascularization survived, whereas no patient underwent PCI or CABG in the mortality group (*P* = .012). The treatments after the onset of PMI were analyzed, including the administration of β-adrenoceptor antagonists, statins, nitrates, anticoagulants, calcium antagonists, diuretics, angiotensin-converting enzyme inhibitors, and angiotensin receptor blockers, and whether the patients underwent PCI or CABG. A total of 15 patients were treated with statins after the onset of PMI: Seven patients in the survival group and eight in the mortality group (*P* = .026). Further, 16 patients were treated with nitrates: 10 in the survival group and 6 in the mortality group (*P* < .001). A total of 20 patients were treated in the ICU, 14 of whom died. The mean ICU stay was 5.13 days (range: 1–71 days) (Table 3). No differences were observed in terms of comorbidities, intraoperative assessments, and preoperative medication (Table 3).

**Table 3:**
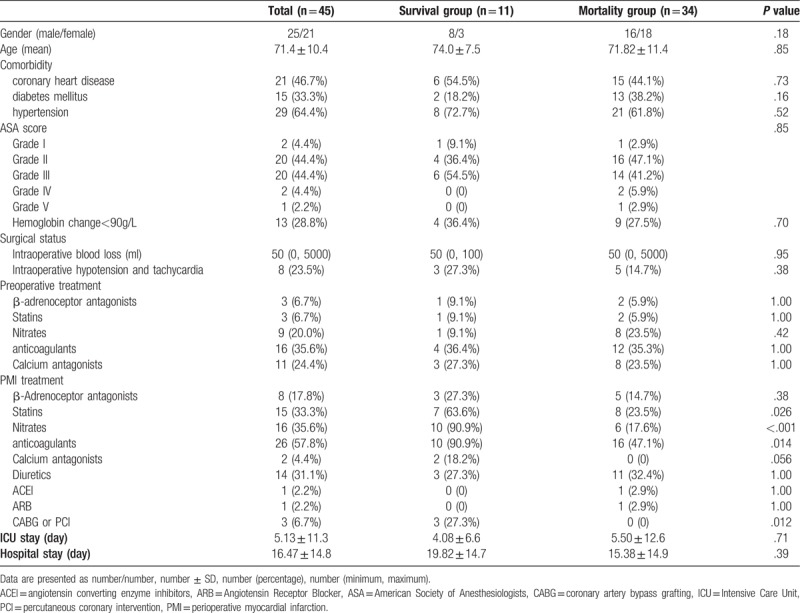
present on potential risk factors of mortality.

## 4 Discussion

Cardiovascular complications are the major contributors to morbidity and mortality after non-cardiac surgery, particularly after PMI (Table 4).^[4–7]^ PMI incidence rates varied widely because of different types of study, different definitions, and different kinds of surgery. A prospective study reported a 7.0% (27/385) PMI incidence rate in patients aged 50 years or older after undergoing non-cardiac surgery.^[7]^ Devereaux et al reported that 5.0% (415/8351) of patients aged 45 years or older had a PMI after undergoing non-cardiac surgery.^[8]^ In a retrospective study, Bangalore et al reported a 0.9% AMI occurrence in patients aged 45 years or older undergoing major non-cardiac surgery in the United States from 2005 to 2013.^[9]^ Menendez et al reported that PMI incidence rates were 0.25% for total hip arthroplasty and 0.18% for total knee arthroplasty in the United States.^[10]^ Li et al reported an overall PMI incidence rate of 5.2 per 10,000 following non-cardiac surgery in a retrospective study.^[6]^ In the present study, the incidence of PMI is 1.61 per 10,000, which is much lower than that in the above-mentioned studies.

**Table 4:**
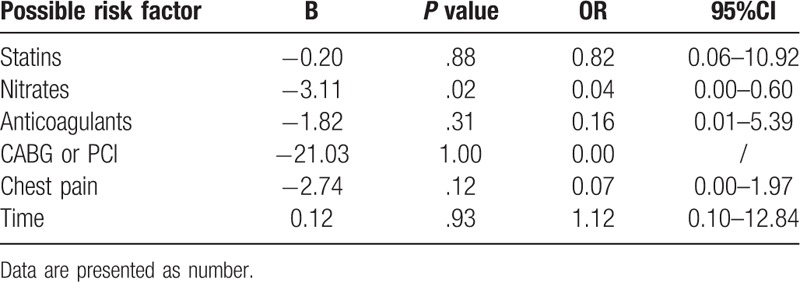
Risk factor of mortality after PMI.

Many factors contribute to the lower morbidity. In this study, we found that PMI occurs mainly within 48 h of surgery and most cases had an onset in the general wards. Unlike in the ICU, ECG monitoring in the general wards usually lasts no more than 24 h in our hospital. Moreover, only 10 patients in this study had chest pain, whereas most PMI cases were asymptomatic. And 100% patients had an onset within 48 h of surgery in the survival group; the onset of PMI was detected later in the mortality group than in the survival group. All the aspects mentioned above indicate the delay of diagnosis or even undiagnosed PMI for many mild or no significant clinical symptoms. Considering the risk factor of PMI, for an earlier diagnosis of myocardial injury, regularly testing troponin T and ECG monitoring for at least 48 h after surgery are necessary for patients who have risk factors of myocardial injury such older than 75 years, underwent vascular surgery,^[9]^ or with history of MI.^[11]^

The treatment of PMI is still not standardized. The therapeutic principles are different between type 1 and type 2 PMI. The pathophysiology of PMI mainly includes the following:

1. the rupture of atherosclerotic plaques and the formation of a thrombus and

2. imbalance between myocardial oxygen delivery and consumption.^[12]^

The two different mechanisms cause two different types of PMI: type 1 (plaque rupture, STEMI) and type 2 (supply–demand imbalance of oxygen; NSTEMI).^[13,14]^ In fact, differentiating between type 1 and type 2 PMI is difficult; it requires a set of diagnostic hints. In the present study, ECG changes were used to identify the differences (sometimes, NSTEMI can change to STEMI). Unlike spontaneous MI, PMI occurred during the perioperative period when patients tended to be under a state of stress. A series of perioperative factors (for instance, tense mood, anesthetic interference, intraoperative blood loss, trauma, and postoperative pain) can increase the myocardial oxygen demand and reduce the oxygen supply, particularly in patients with a history of coronary artery disease.^[1]^ STEMI indicates that one branch (or several branches) of the coronary artery is completely blocked, which requires recanalization.^[15]^ Owing to the risk of bleeding, thrombolytic drugs are not the best choice for patients who have just undergone non-cardiac surgery. A recent study found that the mortality of PMI increased remarkably after PCI^[16]^ as adverse events (e.g., bleeding event and renal dysfunction) cannot be neglected. Regarding NSTEMI, its treatment measures consist mainly of anti-ischemic treatment, anticoagulation/antiplatelet administration, and invasive treatments (PCI/CABG) based on risk stratification. In this study, two patients recovered after undergoing CABG and one recovered after undergoing PCI. In addition, two patients were confirmed to have indications of PCI after consulting a cardiologist; however, the suggestion was refused. As for the use of nitrates, our results show that nitrates are an independent factor for the outcomes of patients with PMI. A meta-analysis that included 27 randomized controlled trials (8244 participants) showed that although more events of cardiac ischemia were observed in participants receiving no treatment or placebo, no statistically significant differences were noted in any comparisons between placebo and nitrates.^[17]^ However, nicorandil has a potential dose-dependent protective effect for cardiac ischemia. But we did not distinguish between different nitrates (nitroglycerin, isosorbide dinitrate, nicorandil, etc) in this study.

There are certain limitations of the present study. First, this was a retrospective single-center study, with weaker power than cohort studies and randomized controlled trials. Second, diagnostic codes were used to screen medical records, and although the authors tried their best to review the medical records, it was difficult to avoid missing cases.

In conclusion, the incidence of PMI in non-cardiac surgery is approximately 2 of 10,000 in patients aged 45 years or older, and increased significantly with age. The use of nitrates might be helpful for their survival.

## Acknowledgments

This study was supported by grants from the National Natural Science Foundation of China (No. 81571030, 81771133), Shanghai Pudong New Area Municipal Commission of Health and Family Planning Funding (PW2016D-4), Shanghai Jiao Tong University integration founding of Medicine and Engineering (YG2017MS53), Shanghai Shenkang Hospital Development Center Founding (SHDC12017X11), and Shanghai Municipal Commission of Health and Family Planning Funding for Key Developing Disciplines (2015ZB0101).

## Author contributions

**Data curation:** Mengyun Tu.

**Project administration:** Zhiying Pan, Diansan Su.

**Software:** Zhongxue Su, Yue Li.

**Writing – original draft:** Xiaoxin Zhou, Lingke Chen.

**Writing – review & editing:** Jie Xiao.

Diansan Su orcid: 0000-0001-9755-1025.

## Supplementary Material

Supplemental Digital Content
