# Supplementary material for: Perioperative acute myocardial infarction in patients after non-cardiac surgery in China: Characteristics and risk factors
Source: Medicine (Baltimore). 2019 Aug 23;98(34):e16929. doi: 10.1097/MD.0000000000016929 (PMC6716711; doi:10.1097/MD.0000000000016929)

Supplementary figure 1. The screening process of patients with PMI

PMI, perioperative myocardial infarction.


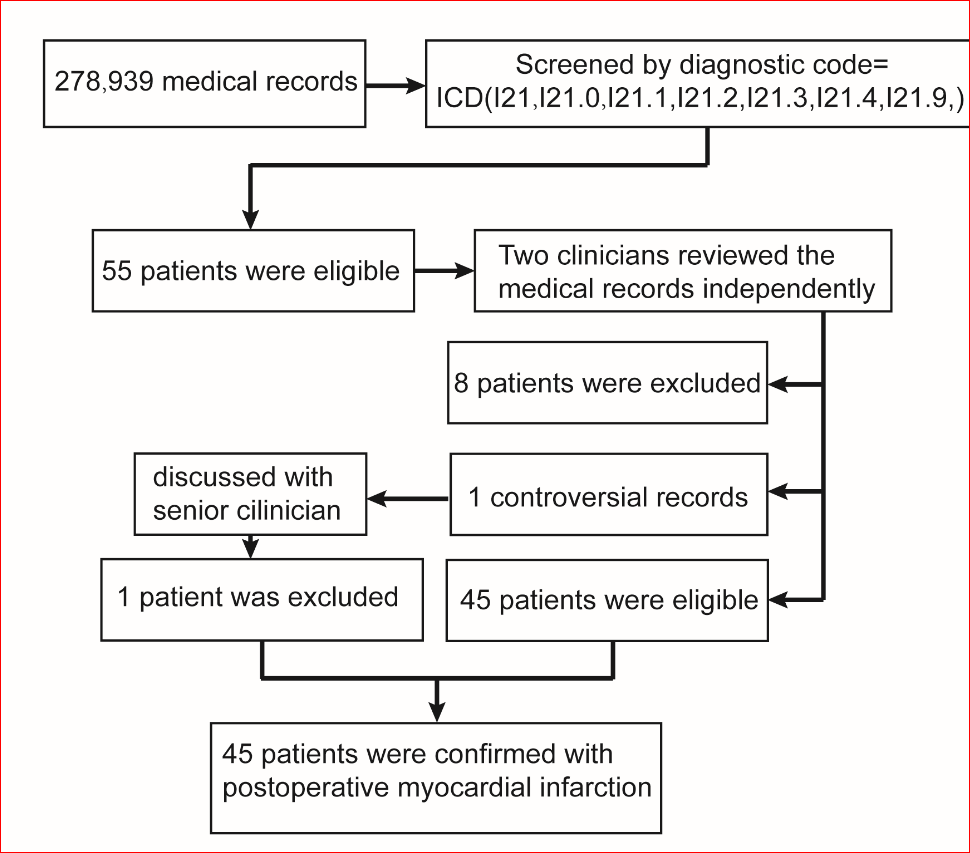

Supplement: Supplemental Digital Content [file medi-98-e16929-s001.doc]
